# Periodic Density Functional Theory Investigation of the Uranyl Ion Sorption on Three Mineral Surfaces: A Comparative Study

**DOI:** 10.3390/ijms10062633

**Published:** 2009-06-04

**Authors:** Jérôme Roques, Edouard Veilly, Eric Simoni

**Affiliations:** Université Paris-Sud 11, Institut de Physique Nucléaire, IPNO bât 100, UMR 8608, F-91406 Orsay, France; E-Mails: veilly@ipno.in2p3.fr (E.V.); simoni@ipno.in2p3.fr (E.S.)

**Keywords:** surface, adsorption, water interaction, uranyl adsorption, DFT, sorption

## Abstract

Canister integrity and radionuclides retention is of prime importance for assessing the long term safety of nuclear waste stored in engineered geologic depositories. A comparative investigation of the interaction of uranyl ion with three different mineral surfaces has thus been undertaken in order to point out the influence of surface composition on the adsorption mechanism(s). Periodic DFT calculations using plane waves basis sets with the GGA formalism were performed on the TiO_2_(110), Al(OH)_3_(001) and Ni(111) surfaces. This study has clearly shown that three parameters play an important role in the uranyl adsorption mechanism: the solvent (H_2_O) distribution at the interface, the nature of the adsorption site and finally, the surface atoms’ protonation state.

## 1. Introduction

Knowledge and understanding about radionuclide retention processes are required for the safety assessment of nuclear waste repository systems. During the transport, radionuclides may be retained by adsorption on highly reactive and divided materials, or transported as dissolved ions or complexes, or in some cases as colloids. Minerals are accordingly important for radionuclide retention in the deep geological repository concepts for high level waste and spent fuel [1,2]. On the other side, the water chemistry in nuclear power plants has been the subject of several studies for many years, because of its influence on the materials degradation, the fuel failures and the radiation fields as well [3]. Materials degradation has to be frequently checked since some components of the plant are very difficult to repair and potentially life limiting. The risk of fuel failures could increase due to heat transfers and the corrosion of the fuel cladding, which depend on the purity of water. Considering a severe reactor accident scenario, corrosion products located near the reactor core could be released into the coolant system. Nickel compounds could be found among the corrosion products in the primary circuit of PWR (Pressurized Water Reactor). Colloidal suspensions could be deposited on pipe walls [4] or float in water. Ni-based corrosion products can have retention properties relative to radionuclides present in the coolant circuit.

In this study, uranium was selected as adsorbate, existing as uranyl cation UO_2_^2+^ (uranium in formal oxidation state +VI) in aqueous solution and acidic pH conditions (between 3 and 4) [5]. In this investigation we propose to describe uranyl interaction with three chemically and structurally different substrates: a transition metal oxide TiO_2_, an aluminum hydroxide Al(OH)_3_, and a transition metal (Ni), using periodic DFT (Density Functional Theory) calculations. Both rutile TiO_2_ and gibbsite Al(OH)_3_ were used here: i) as methodological surfaces and ii) because many experimental data on adsorption process are available. Once the theoretical methodology was established, it will be performed on Ni substrate. Since no retention experiments have been carried out on this system, this particular study will point out the predictive aspect of the presented work.

From a theoretical point of view, very few works have been devoted to studying the interaction(s) of uranyl on mineral surfaces. Moskaleva *et al.* [6] examined uranyl adsorption on the hydrated α-Al_2_O_3_(0001) surface. An outer-sphere adsorption mechanism seemed favored on the fully protonated surface, but no experimental data could be correlated with this result. DFT calculations were also performed with respect to uranyl surface complexation on goetithe (α-FeOOH) and combined with EXAFS (Extended X-ray Absorption Fine Structure) data to validate the surface complex structure [7]. Bidentate uranyl complexes could form on edge and corner surfaces (however, EXAFS measurements were not able to identify the second complex), allowing explanation of the retention properties of goethite. Molecular dynamics studies were also carried out to describe uranyl’s interaction with quartz (010) [8]. Both inner-sphere and outer-sphere complexes were detected, depending on the protonation state of the surface. Last, Monte Carlo simulations were achieved to characterize uranyl surface complexes on montmorillonite [9,10]. The uranyl cation adsorption configuration depended on the charge sites defined in the interlayer sheet surfaces, and also on the water interlayer concentration. For all cases uranyl outer-sphere complexes were suggested to adsorb on the surfaces.

In the case of the TiO_2_ rutile phase, the (110) surface represents 60% of the natural powder (20% represents the (101) surface and 20% the (100) one), and is stable over a large range of pH conditions [11–14]. As it was already demonstrated [5], the (110) face can be used as model of the rutile powder reactivity towards aqueous uranyl ion. Indeed, the U(VI) / rutile TiO_2_ (110) has previously been experimentally studied using various techniques such as EXAFS [15], SSHG (Surface Second Harmonic Generation) [16], AFM (Atomic Force Microscope) and DRIFT (Diffuse Reflectance Infrared Fourier Transform) [5]. The EXAFS data showed that the uranyl ion adsorbed with an inner-sphere mechanism leading to a bidentate surface complex, without any cluster formation upon the TiO_2_ (110) surface. Sorption edges on powder and also single-crystal faces [5] displayed that the adsorption rate increased with respect to the pH (from 1.5 to 4.5). TRLFS (Time Resolved Laser Fluorescence Spectroscopy) measurements [17] advanced the existence of two surface complexes with a relative ratio depending on the pH value. Unfortunately, this technique had not allowed one to deduce the number of adsorption sites and the accurate configuration of the surface complexes. However, regarding the speciation diagram of the uranium ion and since the experiments were performed in the 1.5–4.5 pH range, the uranyl ion is the major species in solution, as aforementioned. These two surface complexes thus corresponded to uranyl cations interacting with two distinct adsorption sites. The AFM experiments have shown that the single crystal face was composed of large terraces (around 100 Å width) of clean surface. As a final point, the DRIFT and the SSHG experiments as well as the modeling of the sorption edges have allowed one to characterize the oxygen surface types involved in the interaction with the uranyl ion.

Hiemstra and co-workers [18–22] and Jodin *et al.* [23] showed that the (001) faces of gibbsite (corresponding to the basal faces) only exhibited uncharged OH groups bonded to two Al atoms [Al_2_(OH)] for pH values ranging between 4 and 10. On the other side, edge faces (corresponding to lateral faces) exhibited both doubly and singly coordinated hydroxyl groups (Al-OH), and possessed a lower surface area than the basal (001) face. In addition, edge faces are positively charged for pH values lower than eight. Consequently, the adsorption of uranyl on gibbsite was investigated on the (001) basal faces due to the acidic conditions in this present study. TRLFS studies [24,25] revealed that at last two kinds of uranyl complexes on the basal surface could be considered to explain the experimental results: a type of ‘Al-UO_2_^2+^’ complex at low pH, and a polynuclear one at higher pH values. However, the nature and the composition of the adsorbed complexes were not entirely described. The formation of an amorphous aluminum hydroxide during adsorption experiments had not allowed to assign any components of the EXAFS and TRLFS studies of Froideval *et al.* [26]; hence surface complexes were not identified as well. Hongxia *et al.* [27] have shown that the insensitive effect of the ionic strength on uranyl adsorption on Al_2_O_3_ and Al(OH)_3_ could be interpreted as the formation of strong chemical bonds between the adsorbed species and the corresponding surfaces, and could be the result of the formation of inner-sphere complexes on the surface.

Concerning uranyl adsorption on the hydrated Ni(111) surface, neither experimental nor theoretical studies have ever been carried out yet, to our knowledge.

In this paper, periodic density functional theory (DFT) calculations were carried out to investigate the adsorption of the UO_2_^2+^ cation on three different hydrated surfaces: the (110) surface of TiO_2_ rutile phase, the (001) surface of Al(OH)_3_ gibbsite phase, and the Ni(111) metallic surface. In a first step, bulk parameters were optimized and compared with experimental data, in order to build accurate surface models. In a second step, water adsorption was investigated in order to obtain the water distribution at the interface and to depict the type of formed interactions. Finally, uranyl adsorption was investigated on the various hydrated surfaces with the aim of describing the created complexes, with respect to the mineral surface.

## 2. Computational Details

In this work, all calculations were performed with the VASP 4.6 code using periodic DFT methodology [28–31]. The GGA (Generalized Gradient Approximation), as defined by Perdew and Wang for exchange-correlation energy evaluation, was used (PW91) [32,33]. All atoms were described with pseudopotentials developed on plane wave basis sets generated with the PAW (Projector Augmented Wave) method [34,35]. Uranium atom was described with fourteen valence electrons (*6s^2^ 6p^6^ 7s^2^ 5f 3 6d^1^*). Spin polarized calculations were carried out. The geometry optimization was performed using the conjugate gradient optimization scheme. The convergence criterions for the self-consistency cycle and the maximum forces were set at 10^−4^ eV and 0.001 eV.Å^−1^, respectively.

### 2.1. TiO_2_ bulk

The titanium atoms were described with four valence electrons (*4s^2^ 3d^2^*) and the oxygen atoms with six ones (*2s^2^ 2p^4^*). This study started by optimizing the rutile bulk parameters in order to determine the accuracy of the modeling by comparing calculated parameters to experimental ones [36]. Calculations were performed using different sets of k-points and energy cutoff to evaluate their effects on the bulk parameters and to optimize them. The bulk rutile unit cell is tetragonal [37] (a=b=4.587 Å, c=2.954 Å, internal parameter x=0.305 and c/a=0.644), and has the space group No 136. Titanium atoms were in 2a positions and O atoms in 4f positions using Wyckoff’s notations. The primitive cell contained two TiO_2_ units. The optimised parameters were obtained with a 5×5×5 k-point meshes and a 350 eV cutoff. These calculated parameters (a=b=4.649 Å, c=2.972 Å, internal parameter x=0.304 and c/a=0.640) are close to the experimental ones and agree with previous theoretical works [37]. They have thus been used to build the TiO_2_(110) model.

### 2.2. Al(OH)_3_ bulk

The pseudopotentials for Al atoms had as valence electrons (*3s^2^ 3p^1^*) and oxygen atoms (*2s^2^ 2p^4^*). Gibbsite has a sheet structure, crystallizing usually in pseudo-hexagonal platelets with monoclinic symmetry. All of the Al(OH)_3_ polymorphs were composed of layers of aluminum atoms located in the 2/3^rd^ of the octahedral sites with hydroxyl groups on either side, half of these groups bonding the layers together. The stacking structures of gibbsite and bayerite were thoroughly described by Giese [38], and Saalfeld *et al.* [39] refined the crystal structure in the centrosymmetric space group P2_1_/n (C_2h_^5^) from XRD (X-Ray Diffraction) patterns. Their results and assignments were recently confirmed by Mercury *et al.* [40]. Starting from the experimental parameters of Saalfeld *et al.* [39], the cell energy convergence with respect to the cutoff energy and the k points sampling was first checked. Using an optimized k points sampling of (2×4×2) and a cutoff energy of 450 eV, fine cell parameters were obtained in comparison with experimental and previous theoretical works. Results after optimization were summarized in Table 1. These optimized parameters were used in the following.

### 2.3. Ni bulk

Nickel adopts a face-centered cubic structure with a lattice parameter a of 3.52 Å, a magnetic atomic moment μ_Ni_ of 0.61 μ_B_, and a bulk modulus B of 186 GPa [43]. Calculations performed on this structure led to the selection of a 12×12×12 k-points grid and a 400 eV cut-off energy. The calculated parameters [44] (a=3.52 Å; μ_Ni_=0.61 μ_B_; B=195 GPa) were in good agreement with the experimental data and with previous DFT-GGA calculations (a=3.53 Å; μ_Ni_ =0.61 μ_B_ ; B=195 GPa) [45].

## 3. H_2_O Interaction with Mineral Surfaces

In this second part, a theoretical study was performed in order to identify the surface sites reactive towards water, and also to make clear if the interface should be considered as a hydroxylated surface (with adsorbed H_2_O and/or OH groups) or a hydrated surface with electrostatic water interactions. This first step will lead to a better understanding of the surfaces/water interface, in order to further study the uranyl adsorption process on these substrates.

### 3.1. H_2_O / TiO_2_(111)

**Figure 1.**
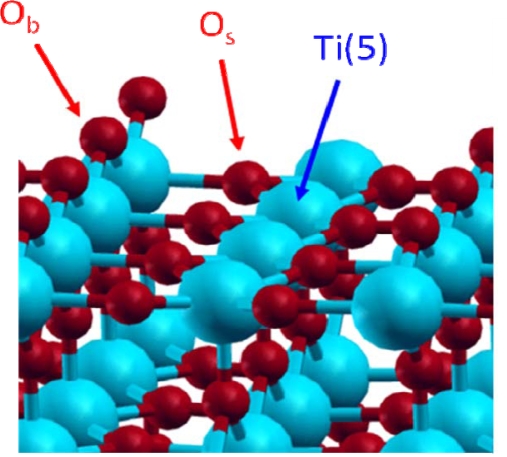
Dry TiO_2_ rutile (110) face.

Among the low index faces, naturally present in the rutile phase powder, the (110) face (60% of the major crystallographic faces) was found as the most stable one [11–14]. For that reason, this face has been chosen in this study. It exhibits titanium and oxygen atoms with different environments (Figure 1). First, a pentacoordinated titanium atom, noted Ti(5), with an unsaturated valence and known as a Lewis acidic site. There are also two kinds of oxygen atoms, the first one is localised in the surface plane and is threefold coordinated (noted O_s_ for “surface” oxygen); the second is prominent from the surface by about 1 Å and is doubly coordinated (noted O_b_ for “bridging” oxygen) and can be considered as a Lewis basic site.

Before studying the water adsorption process itself, the dry TiO_2_ rutile (110) face was optimised with the aim to minimise its size while keeping an accurate description of the system. Internal constraints, consisting in internal layers frozen into their atomic bulk positions, were added in order to stabilise the surface energy for relatively thin systems that allowed working on large surfaces [36]. This led to the choice of a five layer system, with the most internal layer frozen to bulk positions. The cell parameters are: a=13.15 Å, b=26.30 Å and c=8.92 Å corresponding to 60 TiO_2_ patterns. A 10 Å vacuum was created upon the surface in order to avoid any steric interaction between the adsorbed molecule and the upper cell. This accurate TiO_2_ rutile (110) surface model was used to further study adsorption processes [46].

**Figure 2.**
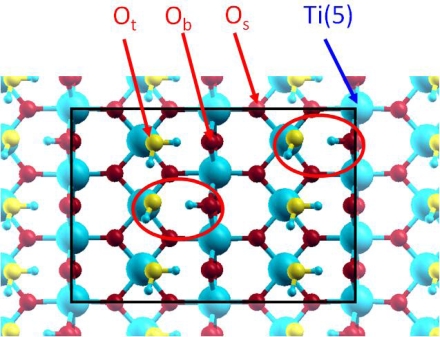
Top view of the 2×3 supercell for the 4 / 2 case. The oxygen atoms of the added water molecules (O_t_) are displayed in yellow colour for clarity.

It is generally considered [47] that water molecule adsorption can occur following two mechanisms: a molecular one, where the water molecule is linked to the Ti(5) atom, which corresponds to the experimental “terminal” oxygen (O_t_) atom doubly protonated; and a dissociative one, where an OH group is linked to the Ti(5) atom, corresponding to the experimental O_t_ atom singly protonated, and the remaining hydrogen atom is transferred to a neighbouring O_b_ atom. For each mechanism, the water molecule, or the two fragments, can be involved in hydrogen bonding (Figure 2).

In the case of a full water coverage (one H_2_O on each Ti(5) for the molecular adsorption case, or one OH on each Ti(5) and one H one each O_b_ for the dissociative adsorption case), the molecular adsorption was energetically the most stable (E_ads_(H_2_O)=−1.04 eV and E_ads_(OH+H)=−0.91eV) which was in agreement with the calculations of Lindan *et al.* [48] who found an adsorption energy of −0.99 eV for the molecular case. However, the dissociative adsorption can also be envisaged because it could be stabilised with hydrogen bonding. Therefore, the partial dissociation of the first hydration layer was investigated with different ratios of molecular/dissociated water molecules. These simulations were performed in order to investigate if molecular and dissociated water molecules could coexist on the surface.

In the following calculations, a 2×3 surface area was used (Figure 2) to study the partial dissociation of the first hydration layer. The supercell was composed of two hundred atoms, leading to a very big supercell. These calculations were performed at the Γ point; the large supercell’s dimension fully justified this choice. The supercell with the six molecular water molecules (the most stable structure) was taken as reference energy. Then, the six water molecules were progressively dissociated. Only the most stable structures were presented in Table 2, where:

- The average destabilisation energy due to the partial dissociation was calculated using Equation (1):
  (1) $$E_{\mathrm{destab}}^{\mathrm{average}}=\frac{E_{\mathrm{ref}}-E_{\text{sup}\mathrm{ercell}}}{N_{H_{2}O}^{\mathrm{dissociated}}},$$
  Where $E_{\mathrm{destab}}^{\mathrm{average}}$ was the destabilisation energy per dissociated water molecule, *E_ref_* was the total energy of the supercell with the six molecular water molecules adsorbed on the six Ti(5) atoms, *E*_sup_ *_ercell_* was the total energy of the considered supercell and $N_{H_{2}O}^{\mathrm{dissociated}}$ was the number of dissociated water molecules.
- The destabilisation of each dissociated water molecule can also be calculated using Equation (2):
  (2) $$E_{\mathrm{destab}}^{n}=E_{\text{sup}\mathrm{ercell}}^{n-1}-E_{\text{sup}\mathrm{ercell}}^{n},$$
  Where $E_{\mathrm{destab}}^{n}$ was the destabilisation energy due to the *n^th^* dissociation, $E_{\text{sup}\mathrm{ercell}}^{n-1}$ was the total energy of the supercell with (*n*−1) dissociated water molecules and $E_{\text{sup}\mathrm{ercell}}^{n}$ was the total energy of the supercell with *n* dissociated water molecules.

Among the six supercells with dissociated water molecules, the smallest destabilization per dissociated water molecule was observed for the 4/2 case. In this structure, two dissociated water molecules were stabilised with hydrogen bonding with four molecular ones, the total destabilization was only −0.04 eV relative to the reference; it corresponded to an average destabilisation of −0.02 eV per molecule. The destabilization energy obtained on the 5/1 system was also relatively weak (−0.03 eV). These calculations revealed that up to 33 % of dissociated water molecules, the destabilization due to water dissociation was significantly compensated by hydrogen bonds (Figure 2).

This result was in agreement with experimental data where characteristic infra-red bands were detected and attributed to surface hydroxyl groups [49,50]. Moreover, some authors [51] have estimated to 25 % the ratio of dissociated water molecules in the first hydration layer, which remained consistent with the calculated range.

### 3.2. H_2_O / γ-Al(OH)_3_ (001)

**Figure 3.**
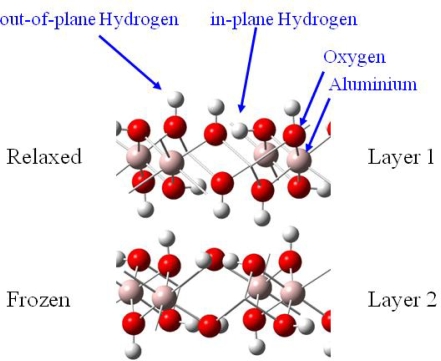
Thetwo-layer gibbsite model used for all calculations.

Uranyl adsorption was assumed to preferentially occur on the basal (001) face of gibbsite, since anion adsorption has been shown to be specific to the lateral faces [18]. In addition, the absence of cation adsorption on edges was coherent with the fact that in the pH range 4–10, the basal faces are uncharged while the lateral ones are positively charged [18,52,53]. The infrared spectra of uranyl adsorbed on the gibbsite sample confirmed this assumption: no change in the lateral OH infrared absorption was observed, whereas changes were reported with sulfate or carboxylate anions [53], in particular the broad band around 3,460 cm^−1^, sensitive to anion adsorption, remained unchanged when uranyl was adsorbed. Consequently, calculations were performed on the (001) face, which exhibits OH groups bicoordinated to aluminum atoms without any deprotonation effects (due to acidic pH conditions), hence corresponding to the crystallographic structure of gibbsite. In order to model adsorption reaction on this surface, it was necessary to evaluate the number of Al(OH)_3_ layers required to reproduce correctly surface relaxation. Surface energies calculations as a function of the number of layer (stacked in the c crystallographic direction) and atomic relaxation localization allowed us to define a two-layer cell [41], with a frozen layer and relaxed one, as an accurate model to study adsorption reactions on the (001) surface (Figure 3).

**Figure 4.**
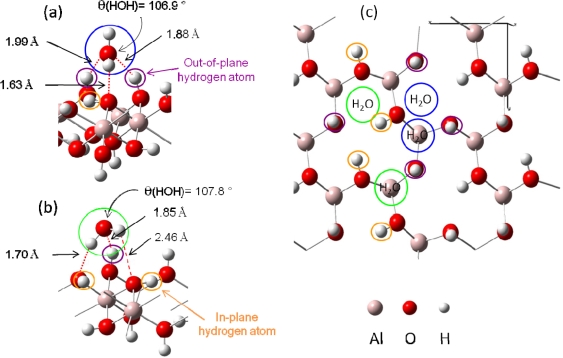
An example of the two possible water adsorption structures (after optimization) is presented on a simplified scheme in panel (a) and (b). Hydrogen bonds are displayed in red color with distances. (a): In blue color, water molecule is linked with two out of plane surface hydrogen atoms and one surface oxygen atom. (b): In green color, water molecule is linked with only one out of plane surface hydrogen atom and two surface oxygen atoms. (c): Top view of the four kinds of H_2_O adsorption sites.

Knowing that H_2_O adsorption on gibbsite took place thanks to a hydrogen bonding network [54,55], interfacial interactions were considered with a maximum of three hydrogen bonds per water molecule (surface sites were defined as a three O-H groups arrangement, with varied hydrogen atoms orientations (in-plane and / or out-of-plane, see Figure 3)). Because of the inner gibbsite (001) surface structure, a surface site could create one or two hydrogen bonds towards the oxygen atom of the water molecule. On the other hand, the water molecule could participate to the interaction sharing one or two hydrogen atoms with surface oxygen atoms [Figures 4 (a) and (b)].

Based on these two adsorption structures, four potential types of sites were considered. Indeed, the presence of one or two out-of-plane hydrogen atoms and the possible presence of one aluminum atom beneath the adsorption site (due to the 2/3 occupancy of the octahedral sites with aluminum atoms) should differentiate the adsorption structures [Figure 4 (c)].

#### 3.2.1. Adsorption at low water coverage

Water adsorption energies at low coverage were first performed for sites composed with two out-of-plane hydroxyl groups [Figure 4 (a)]. Five possibilities for this configuration were found on the whole gibbsite (001) face: two of them involved the presence of a sub-layer aluminum atom, and three others without this latter. Calculations showed that adsorption energies were very similar whatever the surface site (E_ads_ ranging from −0.58 to −0.64 eV), leading to the conclusion that sub-layer aluminum atoms didn’t exert any significant influence on the water adsorption energies. Secondly, water adsorption energies were calculated for sites exhibiting only one out-of-plane hydrogen atom [Figure 4 (b)]. Adsorption energies for the six different possible configurations were found ranging between −0.29 and −0.54 eV. Figures 4 (a) and (b) display the two most stable water adsorption configurations for the two types of site:

- Figure 4 (a) corresponds to adsorption on a “two out-of-plane hydrogen site”, with E_ads_ =−0.64 eV. Distances are the following: d(H_H2O_−O_surface_)=1.63 Å, d(O_H2O_−H_surface_)=1.88 – 1.99 Å.
- Figure 4 (b) shows the adsorption configuration on a “one in-plane hydrogen site”, with E_ads_=−0.54 eV. Water molecule is linked to the surface with two strong hydrogen bonds (d(H_H2O_−O_surface_)=1.70 Å and d(O_H2O_−H_surface_)=1.85 Å) and a weaker third one (d(H_H2O_−O_surface_)= 2.46 Å).

From these results two different orientations of water molecules upon the surface were pointed out. For sites with two out-of-plane hydrogen atoms, the plane of the water molecule was perpendicular to the surface and three hydrogen bonds were created, whereas the molecular plane was almost parallel to the surface for sites exhibiting two in-plane hydrogen atoms. During the optimization process, in-plane OH groups didn’t tilt to create hydrogen bonding with water molecules.

#### 3.2.2. Adsorption at high water coverage

**Figure 5.**
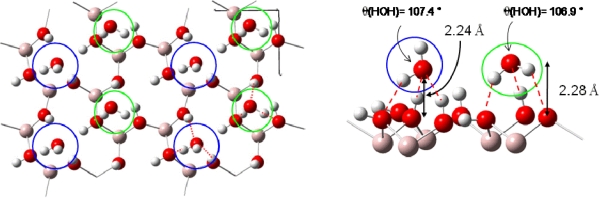
Example of a water monolayer on the (001) gibbsite face. Hydrogen bonds are displayed in red color with distances.

Starting from the most stable water structure at low coverage (three sites with E_ads_=−0.64 eV) the coverage was increased up to water monolayer formation. Calculations showed [41] that three possible ordered water monolayers could be formed on this face with very close stabilities. One water molecule interacted with two out-of-plane hydrogen atoms and one surface oxygen atom (E_ads_=−0.64 eV, first added water molecule in blue color in Figure 5) and the other one with one out-of-plane hydrogen atom and two surface oxygen atoms (E_ads_=−0.45 eV, second added water molecule in green color in Figure 5). One example of a structure is displayed in Figure 5 where hydrogen bonding is displayed in red color. The destabilization due to the adsorption of the second water molecule to saturate the surface was weak and ranged from about 0.05 to 0.1 eV. This was explained by the rather important water-water distance (for instance a minimal distance of roughly 4.5 Å considering the structure of in Figure 5). The water monolayer exposed relatively strong hydrogen bonds with the surface hydroxyl groups, as for the similar octahedral kaolinite surface [56]. Due to the two adsorption structures, periodic alternation of the water molecule dipolar moment direction therefore took place, either directed towards the surface or away from it, as it was observed by Wang *et al.* [55]. The average distance between the surface plane and the water monolayer was calculated to 2.26 Å (the distances of the two water structures are closed (Δd=0.04 Å) see (Figure 5).

### 3.3. H_2_O / Ni(111)

As for the previous studied surfaces, the Ni(111) face was built using the bulk optimized lattice parameters. The optimized surface model was composed of four layers, with a vacuum of 12 Å in order to avoid interactions between the adsorbed molecule and the upper cell [44]. This model was largely validated through previous studies on nickel, but also on pure transition metals or alloys [57–59]. A small contraction of −0.9 % of the distance between the first and the second layer was calculated, as well as a small expansion of about 0.5 % between the second and third layer. This was in good agreement with previous studies [45,60], which allowed us to validate this four layers model to investigate the water interaction on the Ni(111) face.

**Figure 6.**
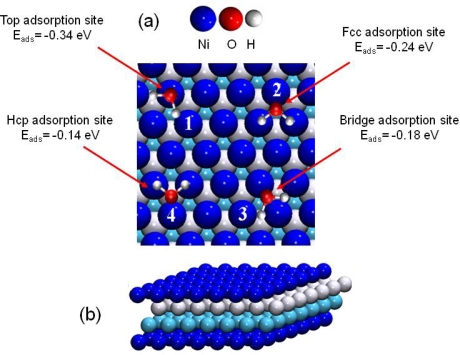
(a) Top view of the water adsorption sites on Ni(111). (1) top, (2) face centered cubic (fcc site), (3) bridge and (4) hexagonal compact (hcp site) adsorption sites. (b) Cut view of the four layer Ni(111) model.

At low coverage (1/9 MonoLayer (ML) coverage), the adsorbed water molecules can be considered as isolated (no hydrogen bonding: d(H_2_O-H_2_O)=7.46 Å). Water adsorption was investigated for each high-symmetry site on the Ni(111) model to determine the most stable adsorption configuration and their relative energy stabilities (Figure 6). The corresponding adsorption energies were calculated according to Equation (3):

(3) $$E_{\text{ads}}=E_{\mathrm{surface}}^{\mathrm{hydrated}}-(E_{{\text{H}}_{2}\text{O}}+E_{\mathrm{surface}}^{\mathrm{dry}})$$

With *E_ads_* the adsorption energy of one water molecule, $E_{\mathrm{surface}}^{\mathrm{hydrated}}$ the energy of the surface model with one adsorbed water molecule, *E_H_2_O_* the energy of an isolated water molecule, and $E_{\mathrm{surface}}^{\mathrm{dry}}$ the energy of the clean Ni(111) face.

Calculated adsorption energies are summarized in Table 3 and compared with previous calculations and experimental data.

The “on top” structure (configuration 1, Figure 6) was calculated as the most stable one with an adsorption energy of E_ads_=−0.34 eV. The corresponding distance between nickel and oxygen atoms was calculated to 2.10 Å. This structure has already been observed and calculated, at low coverage, for water molecules on nickel at higher coverage (¼ ML, [61,65]) and on other transition metals (like Pt(111), Ru(0001), Rh(111), Pd(111), Cu(111), Ag(111) and Au(111) [59,66–68]). The recent work of Li *et al.* [65] has shown that the Ni-OH_2_ bond was principally due to the hybrid orbitals: 3p_z_(Ni)–2p_z_(O), 3p_y_(Ni)–2p_z_(O), 3p_z_(Ni)–2p_y_(O) and 3p_y_(Ni)–2p_y_ (O).

**Figure 7.**
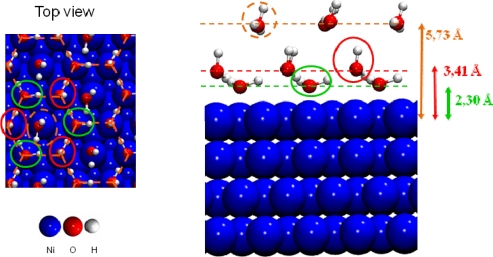
The three water layers on the Ni(111) face.

The water coverage was then progressively increased without considering cluster formation (water dimer). Average adsorption energies per water molecule were calculated as a function of the surface coverage according to Equation (4):

(4) $$E_{\mathrm{ads}}^{\mathrm{average}}=\frac{E_{\mathrm{surface}}^{\mathrm{hydrated}}-n\;E_{{\text{H}}_{2}\text{O}}-E_{\mathrm{surface}}^{\mathrm{dry}}}{n}$$

where, $E_{\mathrm{abs}}^{\mathrm{average}}$ was the average adsorption energy per water molecule, $E_{\mathrm{surface}}^{\mathrm{hydrated}}$ was the total energy of a cell containing the surface with *n* water molecules, *E_H_2_O_* was the energy of an isolated water molecule in vacuum, and $E_{\mathrm{surface}}^{\mathrm{dry}}$ was the energy of the clean surface. Results showed the competition between the stabilization due to hydrogen bonds formation between water molecules and the destabilization resulting of oxygen – oxygen repulsions. Three parts can be separated [44]:

- For surface coverage lower than 2/3 ML, the adsorption energies were in the range of isolated water molecules (≈ −0.3 eV)).
- For 2/3 ML surface coverage distances between water molecules were ∼2.5 Å and hydrogen bonds were created stabilizing the water network. Water molecules were organized in a bilayer H-up-hexamer structure which will be detailed below.
- For higher coverage than 2/3 ML water molecules pushed back themselves, which led to the formation of a third layer. The three water layers are displayed in Figure 7.

Calculations showed that a water saturation occured at 2/3 ML surface coverage, in good agreement with the XPS (X-ray Photoelectron Spectroscopy) study of Pache *et al*. [62]. When the saturation is reached, a cyclic hexamer structure of water molecules took place on the surface (Figure 7). This structure has been proposed to exist experimentally [69] and calculated on several flat transition metal surfaces [67,68]. Each water molecule formed three hydrogen bonds with its water neighbors. Water molecules could be separated in two categories: the first type (called first water layer in this study) was located at 2.30 Å from the surface nickel atoms, forming planes which make a bond angle of 4.58° with the surface. Covalent O–H bonds measure 1.02 Å (0.99 Å for a water molecule at low coverage). Each O–H bond pointed toward a water molecule of the second type. The second type (called second water layer in this study) was located at 3.41 Å from the surface nickel atoms and the H_2_O plane was almost perpendicular to the surface. One hydrogen atom was steered in the opposite side of the surface (d_O–H_ =0.99 Å), and the second one toward an oxygen of a water molecule of the first water layer (d_O–H_ =1.07 Å). The H-O-H bond angle had a value of 107.02°. The calculated structure was in agreement with the angle resolved UPS (Ultraviolet Photoelectron Spectroscopy) spectra showing a splitting of the 3a_1_ level which indicated the existence of two nonequivalent water molecules in the adsorbed bilayer water structure of the hexamer on the clean Ni(111) face [62]. Adsorption energy calculations, per water molecule, revealed that only the water molecules of the first layer interacted directly with the nickel surface (E_ads_≈ −0.20 eV), whereas those of the second layer didn’t interact directly with the surface, but formed hydrogen bonds with the first water layer [44]. The interaction energy of one water molecule of the second layer was estimated to −0.85 eV. As each molecule possessed three hydrogen bonds, the average H bonds strength in the bilayer H-up-hexamer was around −0.28 eV, which was in agreement with the works of Michaelides *et al.* [70] (−0.27 eV on Ru(0001)) and of Meng *et al.* [71] (−0.26 eV on Pt(111)). If additional water molecules were introduced after saturation of the nickel surface at 2/3 ML coverage with the bilayer H-up-hexamers, the formation of a third water layer was observed (Figure 7). It was located at 5.73 Å of the surface. Each water molecule was physisorbed on top of a bilayer H-up-hexamers with an average calculated adsorption energy of −0.33 eV. This results is in agreement with TPD (Temperature Programmed Desorption) measurement [62], showing that after saturation of the α-state corresponding to the water ‘bilayer’ at 2/3 ML coverage, two additional peaks appeared in the spectra upon further increase of exposure. At first the β-state grows in, which was attributed to a transition state between the water ‘bilayer’ and the condensed ice layer which corresponded to the third peak observed. This result suggested that for adsorptions on top of the ‘bilayer’ there was still an effect of the substrate on the water distribution.

### 3.4. Conclusion of the water part

These results clearly put forward a different behaviour of water at the interface of the three studied materials: Concerning oxide metal TiO_2_, the presence of Ti(V) CUS (Coordinatively Unsaturated Sites) on the (110) surface engendered direct adsorption of water molecules to create Ti-OH_2_ groups with an adsorption energy of −1.04 eV for the most stable structure (molecular adsorption). However, water dissociation could take place up to a 33% ratio of proton transfer from adsorbed H_2_O to the neighbouring “bridging” O_b_ atom, in good agreement with experimental data [51]. On the other hand, gibbsite (001) surface exhibited two kinds of O-H groups, depending on the arrangement of the hydrogen atom (perpendicular or parallel to the surface plane). Water interaction consequently took place by means of several weak hydrogen bonds. The first water monolayer evidently involved a mixing of two water adsorption configurations, as already suggested by means of molecular dynamic calculations [55]. Finally, metallic nickel (111) surface incited a particular water arrangement for a 2/3 ML surface coverage (corresponding to saturation). Water molecules were organized in a bilayer H-up-hexamer structure, where hydrogen bonds are created and stabilize the water network. The reactivity of the three substrate towards water being well characterized, the uranyl interaction on the different hydrated surfaces could be investigated.

## 4. Uranyl Interaction on the Hydrated Mineral Surfaces

### 4.1. Uranyl / hydrated-TiO_2_(111)

**Figure 8.**
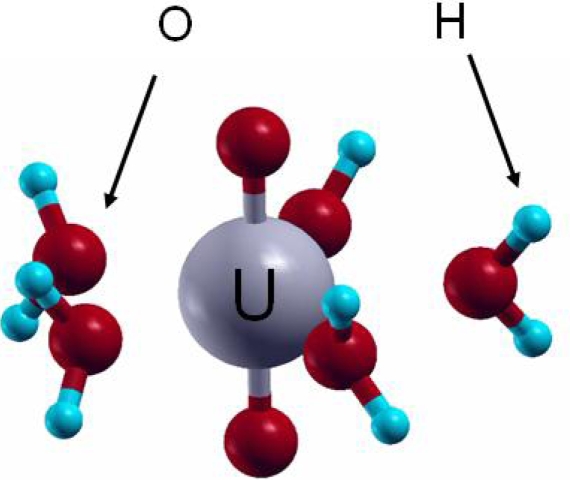
Pentahydrated uranyl structure [UO_2_(H_2_O)_5_]^2+^.

The +VI oxidation state is observed for uranium in aqueous solution under oxidizing conditions. From the aqueous uranium speciation diagram [5], the uranyl ion (UO_2_^2+^) is the main species at pH < 4. Knowing that the free uranyl ion in acidic solution exhibits five water molecules in its first hydration shell ([UO_2_(H_2_O)_5_]^2+^) [15], VASP calculations were performed to assess the geometrical and energetic features of the complex. Calculations were thus carried out on [UO_2_(H_2_O)_n_]^2+^ complexes, with *n* from 0 to 6, with all the water molecules in the first hydration shell of the uranyl ion. For n from 4 to 6, additional structures with water molecules in a second hydration sphere were also tested [72]. In agreement with experimental data and previous calculations, the pentahydrated uranyl ion (Figure 8) was characterized as the most stable complex with d(U=O^-yl^)=1.78 Å (exp: 1.77±0.002 Å) and d(U-OH_2_)_average_=2.46 Å (exp: 2.42±0.02 Å). In addition, the average hydration energy of −2.18 eV calculated for the pentahydrated complex was in agreement with localized PW91 (−2.20 eV) and MP2 (second level Moller Plesset) (−2.10/−2.17 eV) calculations [73–75]. This good correlation between these results and experimental data showed that plane waves periodic DFT calculations, which is not the most appropriate approach to study molecular systems, was on the contrary powerfull to describe molecular structures such as [UO_2_(H_2_O)_5_]^2+^.

Since theoretical results from periodic DFT calculations for the isolated aqueous uranyl ion were in good agreement with experimental data, the interaction between uranyl and the hydrated surface was then investigated using this methodology.

**Figure 9.**
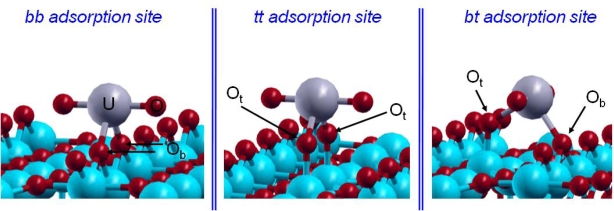
The three studied adsorption sites.

From a crystallographic point of view, two kinds of surface oxygen atoms were supposed to be reactive on the hydrated TiO_2_(110) surface: the bridging and terminal ones (Figure 2). As EXAFS data revealed that the uranyl ion interacts with the TiO_2_(110) face with an inner-sphere mechanism, leading to a bidentate surface complex with no aggregation phenomenon, three different possible bidentate adsorption sites had to be considered (Figure 9): bridging-bridging (noted *bb*), terminal-terminal (noted *tt*) and finally bridging-terminal (noted *bt*).

However, only two adsorption sites were observed experimentally [15] and were attributed to the bridging-bridging and bridging-terminal sites [17]. Calculations [72] were carried out with a supercell surface dimension of 13.2×8.9 Å^2^ and containing up to 200 atoms. On this large surface, the uranyl ion should not interact with its own image (d(U–U)_min_=8.9 Å), in agreement with EXAFS data where no uranium–uranium interactions were detected. To keep its pentadentate equatorial hydration shell, the uranyl ion was saturated with three water molecules. Low pH conditions were simulated by saturating with protons all reactive surface oxygen atoms (terminal and bridging oxygen atoms). The CD-MUSIC model [19,76] allowed us to evaluate the intrinsic *pK* values of the different oxygen surface sites by considering their atomic environment and their saturation. By applying this model to the rutile TiO_2_ (110) face, the behavior of the three oxygen surface species could be defined: the O_s_ atoms were not found to be reactive to protonation; next, the O_b_ ones can be protonated once with a corresponding calculated *pK*_1_ of 4.4; at last, the O_t_ atoms were at least once protonated in aqueous solution and a second protonation was possible with a calculated *pK* _2_ value of 7.5. The third protonation was not possible in aqueous solution. Therefore, as experiments were performed at very low pH conditions (pH=3), all O_t_ atoms were doubly protonated and O_b_ ones only singly, which corresponded to our saturated surface model. No hydrogen was added on oxygen atoms of the adsorption site (it was calculated to be less stable or unstable [72]). The bond lengths in the three optimized structures were compared to the EXAFS results and the relative uranyl adsorption energies were calculated (Table 4).

Regarding the relative adsorption energies of the uranyl ion, it appeared that the *bb* and the *bt* structures were the most stable ones and energetically very close. The third structure, the *tt* one, was 175 meV less stable than the *bb* one, which was in agreement with the experimental result: only two uranyl surface complexes, on the two most reactive adsorption sites (*bb* and *bt*), were observed on the TiO_2_ (110) surface. Among the structures, the *bb* adsorption site was found to be the most stable one in agreement with experimental data [16]. Looking at the U=O and U–O_water_ distances, a part of the lengthening was most likely due to the GGA formalism. However, since GGA was known to give more reliable energies for molecular species than LDA (Local Density Approximation) formalism, it has so been preferentially used in this study. A major part of this lengthening can be linked to the lack of solvent effects as well, not taken into account in these calculations, that should favor the stabilization of the =O and –OH_2_ bonds. Regarding the U–O_surface_ bond lengths, the average distances determined by EXAFS at 2.31 Å were consistent with the average calculated optimized distances. It was also observed that the adsorbed uranyl ion was not linear contrary to the pentahydrated form in solution, as also calculated by Moskaleva *et al.* on hydroxylated alumina surfaces [6]. The observed bending was related to the low bending frequency of the uranyl, calculated by Clavaguéra-Sarrio *et al.* [74] between 100 and 180 cm^−1^ (depending on the exchange-correlation functional used). In addition, XANES (X-ray Absorption Near Edge Spectroscopy) spectrum calculation shown that until a torsion angle of 20 degrees, the characteristic signal of the uranyl ion was still present [15]. Therefore, taking into account this calculation from the experimental XANES spectrum, the O=U=O angle could be in the range of 160–200°.

### 4.2. Uranyl / hydrated-γ-Al(OH)_3_(001)

**Figure 10.**
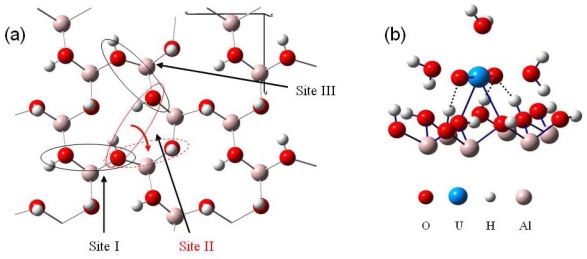
(a) Top view of the (001) gibbsite surface, with the three crystallographic active sites towards uranyl adsorption. (b) Uranyl adsorption structure on site I (monolayer water molecules have been omitted for clarity).

As it was shown that the gibbsite edge faces were strongly positively charged [18,52], while in the pH range of this study (low pH, ranging between 3 and 4) the basal charges may be neglected, it was expected that uranyl cation were adsorbed on the basal faces. This trend was confirmed using infrared spectra. Indeed, the absorption spectra did not show any significant change in the lateral OH stretching region after the adsorption of the uranyl cations in opposition with the anion retention [41,52,53]. Moreover, a bidentate uranyl complex formation (leading to an inner sphere complex) was assumed to be favorable on the (001) gibbsite surface, according to the literature [15,77–79]. Consequently, surface complexes in our computation possessed three water molecules as first hydration shell. The surface basal oxygen atoms of hydroxyl groups with in-plane hydrogen were at first sight good candidates to link with the uranium atom, since they offered a direct possibility of linkage of the uranium atom in the uranyl equatorial plane. Only three potential structural sites of this type were available on the (001) face (see Figure 10), due to the high symmetrical surface configuration: two of them hold roughly the same O-O distances (about 2.7 Å, site I and III), whereas the last one exhibited a longer O-O distance (3.4 Å, site II).

Relative energies and characteristic distances for the three optimized structures were summarized in Table 5 in the *protonated sites* part.

The most stable adsorption configuration corresponding to site II is displayed in Figure 10, panel b. For this site, a shift of uranyl occurred during the optimization. Indeed, due to the important initial O_surface_−O_surface_ distance (3.4 Å) compared to site I and III (2.7 Å), uranyl ion rotated from starting site II (displayed in full red line, Figure 10, panel a) to a neighbored site surrounded in red dashed line. This latter (d(O-O)~2.7 Å, like site I and III) exhibited prior to uranyl interaction, one in-plane and one out-of-plane hydrogen atoms. The out-of-plane hydrogen atom fell over in the surface plane during uranyl adsorption. In a general way, after optimization, only one type of surface complex were calculated: all of them were stabilized throughout a hydrogen bonds network by means of three hydrogen bonds (dashed line in Figure 10, panel b) between the surface and the ‘-yl’ oxygen atoms. One of the O^-yl^ atom hold two hydrogen interactions, whereas the other was single bonded. Distances between uranium atom and surface oxygen atoms were in the range 2.5–2.9 Å, indicating to a rather iono-covalent bonding nature with a relatively strong ionic part. A slight decrease of the O=U=O bond angle occurred when adsorption, reaching a minimum of 168.4° for site III. U-(O^-yl^) distances were slightly increased upon adsorption, about 1.92 Å (average value) for all complexes. Distances between the three first hydration shell water molecules and uranium atom also increased: calculated average values were of 2.57, 2.53 and 2.55 Å for site I, II and III, respectively.

Nevertheless, when the above mentioned adsorption reaction occurred, reactive oxygen atoms of the (001) plane linked to the uranium atoms adopt a rather unsteady four-fold coordination state. A possible local deprotonation of the hydroxyl groups could thus be assumed. Characteristics distances and relative energies of the complexes are given in Table 5 in the *deprotonated sites* part. Geometrical features of this kind of configuration were the following. The averaged U=O distances were similar to the ones observed for protonated complexes, although a little bit shortened (average value: 1.89 Å). A stronger interaction took place between uranyl and the surface due to lower coordination of oxygen atoms. Indeed, the U-O_surface_ distances were decreased of around 0.5 Å compared to surface protonated complexes, plainly revealing a more covalent bond character. The O=U=O bond angles were highly distorted (146.9, 162.9 and 150.8° for sites I, II and III, respectively) compared to protonated complexes, as it was already observed within periodic DFT calculations of uranyl adsorption on hydroxylated alumina [6]. This behaviour is coherent with the decrease of the distance U-O_surf_. The distances between the three remaining water molecules and the uranium atom increased of about 0.1 Å, relative to protonated complexes.

From these last calculations, it can be concluded that even if several complex structures had to be considered primarily, all of them converged to a very similar final complex structure. Therefore, only one type of complex structure was calculated and determined on this gibbsite face (whatever the protonation state of the adsorption site). Indeed, local deprotonation of the surface oxygen atoms involved in bonds was also investigated. A significant decrease of the U-O_surface_ bonds was observed resulting in a most important interaction with the surface. Calculations on a dry (001) gibbsite face were also tested in order to analyze the effect of the surface hydration on the adsorption process. No major changes in the adsorption geometries were detected (for protonated sites as well as nonprotonated ones). Only a slight decrease of the hydrogen bonds lengths between the adsorbed molecule and the surface was noticed (because hydrogen bonds between O^-yl^ and water molecules didn’t exist anymore).

In support to this theoretical approach, Raman and TRLFS experiments [41] were performed in order to check the validity of the DFT results. TRLFS results showed that only one type of uranium adsorption site was present on the (001) gibbsite face at pH=3 and for [U(VI)]=10^−4^ and 10^−3^ M, in perfect agreement with the above theoretical results. Moreover, as the spectroscopic parameters of the adsorbed species were the same at 0.1 M and 1 M ionic strength, the formation of inner-sphere complexes initially assumed for calculations is therefore fully established. Indeed, at a high ionic strength the retention *via* the formation of outer-sphere complexes was strongly unfavorable while the formation of inner-sphere complexes was not influenced at all [80–83]. Raman results confirmed the bidentate adsorption mode of uranyl on the surface. Unfortunately, no experimental data is yet available to know if the local deprotonation of the adsorption site oxygen atoms can take place during the adsorption process. Therefore, EXAFS measurement will be soon performed in our group to get the U-O_surf_ distances in order to answer this question.

### 4.3. Uranyl / hydrated-Ni(111)

As no experimental data have been yet available for this system, we first investigated the uranyl adsorption on the dry Ni(111) face and in a second step on the hydrated one, in order to analyze the solvent effect on the adsorption process. In the case of the hydrated surface, the adsorption with an inner as well as outer sphere mechanism was investigated.

**Figure 11.**
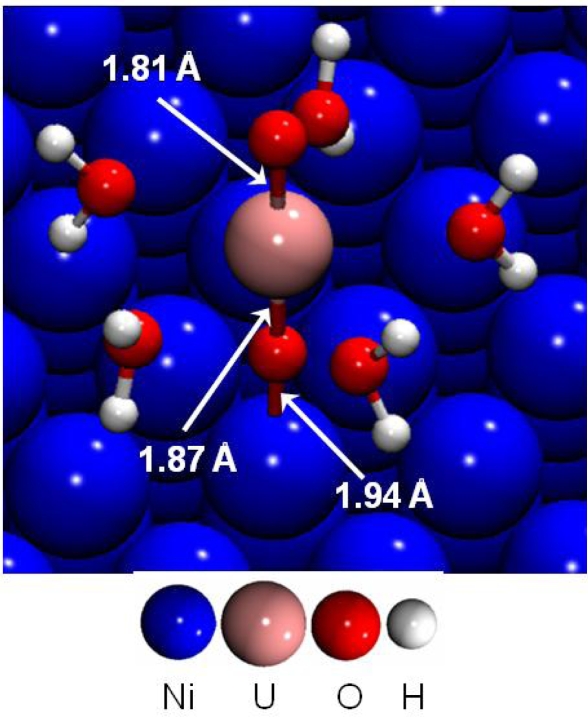
Adsorption of UO_2_(H_2_O)_5_^2+^ on the dry Ni(111) at low coverage.

First, the adsorption of the uranyl ion at low coverage was investigated on the dry (111) nickel surface [44]. The most stable surface complex is presented in Figure 11. Uranyl rod was perpendicular to the surface, the distance d(U-O^-yl^) was calculated to 1.87 Å near the surface and 1.81 Å on the other side. Indeed, all the parallel starting structures with direct H_2_O-Ni interactions were calculated as unstable.

The interaction between the uranyl ion and the nickel surface was therefore made through an O^-yl^ atom and a surface Ni atom. Adsorption energies for the four different calculated stable surface complexes were summarized in Table 6. It was shown that hydrated uranyl ion adsorbed preferentially on top of a surface nickel atom through a Ni-O^-yl^ bond, with a strong adsorption energy of −8.89 eV. This structure represented in Figure 11, displayed a Ni-O^-yl^ bond length of 1.94 Å. The first hydration shell geometry remained almost unchanged compared with aqueous uranyl cation: the five water molecules were optimized at an average distance of 2.52 Å from the uranium atom. Water molecules were at a distance d_Ni-O(-water)_ of 3.95 Å from the nearest nickel atom and the uranium atom at 3.81 Å. To sum up, a strong Ni-O^-yl^ interaction took place between the uranyl and the metallic surface.

**Figure 12.**
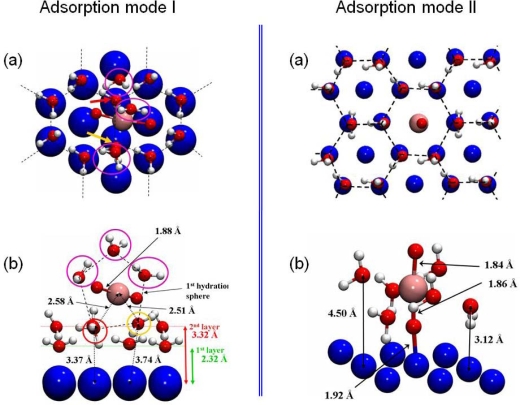
The two adsorption modes of the uranyl ion on the hydrated Ni(111) face were displayed. For each one, (a) represents a top view and (b) a cut view. The third water layer was omitted in these pictures to clarify the view.

The interaction of the uranyl ion was then investigated on the hydrated surface model, optimized in the previous part (3×3 unit cell with four Ni layers). Two structures were calculated as stable and are presented in Figure 12.

For the first structure, the uranyl ion was adsorbed on the surface through its first hydration shell and shares two water molecules with four surface hexamers (left panel of Figure 12). The uranyl hydration shell for this outer-sphere mechanism adsorption mode was composed of five water molecules, as for isolated aqueous uranyl cation. Both U=O bond lengths had a value of 1.88 Å. The uranium atom was almost equidistant and quite far away from the two nearest nickel surface atoms (d_U-Ni_ =5.61 and 5.67 Å), and so didn’t interact directly with the surface. Hence, this adsorption mechanism took place through an hydrogen bonds network with the first hydration shell. The two water molecules (pointed out in yellow and red color in the left part of the Figure 12**)** were shared between the first hydration shell of the uranyl and four hexamers at 2.58 Å and 2.51 Å from the uranium atom (Δd=+0.12 Å / +0.05 Å compared with uranyl ion in aqueous solution). This adsorption mode induced a slight increase of the distance between the uranium atom and its first hydration shell: the average distance between the uranium atom and the three others water molecules went up to 2.70 Å (2.46 Å in gas phase). This last value was surely overestimated due to the lack of solvent effects, as it was previously calculated in prior works [6,41, 79]. Distances between the two water molecules of the uranyl first hydration shell and the nearest Ni surface atoms (3.37 and 3.74 Å (left part of the Figure 12, panel b)) implied that one of the shared water molecule (the chemisorbed one) was pulled out from the surface and no more interacted directly with Ni(111).

The second adsorption configuration was displayed in the right part of Figure 12. The O=U=O axis was perpendicular to the surface, on top of a free surface nickel atom at a distance of d(Ni-O^-yl^)=1.92 Å. The uranyl ion contracted six water molecules in its first hydration shell consequent to this adsorption mode. Accordingly, it led to an increase of the U-OH_2_ distances of 0.21 Å (average value) relative to free uranyl ion (2.46 Å). Although the presence of six water molecules in the uranyl first hydration shell was not the most stable coordination mode in aqueous solution, this structure remained possible: the solvation energy with five water molecules was calculated to E_solvation_=−2.23 eV, which was 0.33 eV more stable than with six water molecules [72]. Besides, comparison with the hexamers without adsorbed uranyl displayed an increase of the distances between the hexamer and the nickel surface atoms (Δd=+1.0±0.2 Å). This demonstrated that when uranyl ion adsorbed perpendicular to the surface, water molecules interacted in a preferential way with the ion instead of with the surface. Because of various interaction components for this kind of adsorption mode (hydrogen bonds, U•••OH_2_, O^-yl^•••Ni, etc …), it was not straightforward to calculate the overall adsorption energy. Therefore, to estimate the O^-yl^•••Ni bond energy, comparison has been done with calculation of the uranyl ion adsorbed on the dry Ni(111) face. In this case, as view previously, the Ni-O^-yl^ bond length was 1.94 Å for a calculated adsorption energy of −8.89 eV. The addition of hexamers on the surface had almost no influence on the Ni-O^-yl^ distance (−1.04 %). Hence, it was concluded that the O^-yl^•••Ni bond energy shouldn’t noticeably change when water hexamers cover the surface. It was also deduced that the main part of the interactions between the surface and the uranyl ion was done through the O^-yl^ atom. The complex was furthermore stabilized through its first hydration shell, which formed hydrogen bonding with surrounding water hexamers which were bonded to the surface.

## 5. Conclusions

Results obtained in this study were relevant to the retention of the uranyl radionuclide on three distinctive mineral surfaces (rutile TiO_2_, gibbsite Al(OH)_3_ and Ni). It was demonstrated that periodic DFT calculations can provide a high level of understanding that is necessary to correctly describe uranyl retention reactions. Results showed that the adsorption behaviour was strongly dependent on the nature of the mineral phase. Calculations were performed using the VASP code in three steps for all substrates.

First, it was necessary to determine the crystallographic parameters for the bulk minerals, in order to check the robustness of this approach and to afterwards model accurate surfaces. These parameters were optimized starting from crystallographic experimental data. To check the validity of the calculated parameters, minerals physical properties were calculated and compared when possible to existing ones found in the literature. Then, the TiO_2_(110), Al(OH)_3_(001) and Ni(111) faces were modelled. To correctly reproduce the surface properties with the smallest model, internal constraints were imposed.

In a second part, since uranyl adsorption takes place at the liquid/solid interface, water interaction on the three surfaces was investigated first. Calculations revealed varied behaviour of water molecules on surfaces. Concerning the TiO_2_ rutile phase, water interaction was found to be strong because the (110) face exhibit CUS in the form of Ti(5) atoms. Thus, Ti-OH_2_ groups were formed, and can be partially dissociated by transferring a hydrogen atom on a neighbouring O_b_ (“bridging” oxygen) atom to create a bridge bonded hydroxyl group. The calculated water dissociative ratio (around 33%) was in good agreement with experimental value. However, because of the experimental acidic condition applied during the adsorption process (pH=3), the surface was fully protonated, consequently exhibiting a mix of (Ti)OH_2_ and (Ti_2_)OH groups in equivalent amount. On the other hand, water interaction on the gibbsite (001) surface led to a quite strong hydrogen bond network, since the surface expose O-H groups. Two water adsorption configurations arose, related to the presence of one or two out-of-plane hydrogen atoms per site on the surface. Monolayer coverage involved a periodic alternation of the two configurations, with adsorption energies of −0.64 eV (site with two out-of-plane hydrogen atoms) and −0.45 eV (site with one out-of-plane hydrogen atom). Finally, water interaction with the metallic Ni(111) surface generated the formation of a bilayer H-up hexamers structure for a coverage of 2/3 ML (matching to surface saturation with water). Two types of water molecules belonging to hexamers were observed: the first layer interacted directly with the surface (E_ads_=−0.2 eV per water molecule), while the second one was bounded by means of hydrogen bonds to the first water layer. If the coverage was still increased, a physisorbed third water layer appeared on top of each hexamer.

Finally, the three hydrated surfaces being fine characterized, the interaction with the uranyl cation was investigated. Before studying uranyl adsorption, one needed to optimize the structure of [UO_2_(H_2_O)_5_]^2+^, the predominant and most stable hydrated complex in acidic aqueous solution. Calculations showed good agreement for the optimized cation geometry with spectroscopic results as well as previous theoretical ones. Then, uranyl interaction with TiO_2_(110) was first considered. Three potential sites were detected on the surface, and two kinds of oxygen atoms (O_t_ ‘terminal’ and O_b_ ‘bridging’) were found to be reactive towards adsorption. Indeed, EXAFS data revealed that uranyl adsorbed *via* an inner sphere mechanism, leading to a bidentate adsorption mode. Therefore, when uranyl adsorbed, two first shell water molecules were removed in order to allow uranium bonding with the surface. Calculated relative adsorption energies confirmed that the *bb* and *bt* structures were the most stable, in accordance with experimental results. If U=O and U-OH_2_ distances were calculated slightly longer than EXAFS ones, due to the partial description of the solvent effect, U-O_surface_ distances matched quite perfectly (2.31 Å). Finally, the linearity of the uranyl disappeared during adsorption. This was in agreement with the XANES spectrum calculation showing that up to a torsion angle of 20 degrees, the characteristic signal of the uranyl ion was still present, which means that the O=U=O angle could be in the range of 160–200°. Regarding to gibbsite (001) surface, three potential crystallographic sites were determined. Indeed, literature allowed one predicting a bidentate adsorption mode upon the (001) surface. Therefore, surface oxygen atoms bearing in-plane hydrogen atoms appeared to be good candidate to link with the uranium atom. Distances between the complexes and the surface, in the range of 2.5–2.9 Å, were characteristic of an ionocovalent interaction composed of a significant ionic part. Calculated U=O distances and U-OH_2_ were around 1.92 Å and 2.53 Å, respectively (average values), that is, longer than for uranyl in the gas phase. The O=U=O bond angle slightly curved due to the adsorption. To sum up, it was observed that all optimized complexes displayed a similar structure, even if various stabilities were detected. In addition, it should be noted that surface site deprotonation was taken into account, because of the instability of the four-fold coordinated oxygen atoms engaged with uranyl molecule. The main geometrical features resulting from oxygen atoms deprotonation were a significant decrease of the U-O_surface_ bond lengths (from 2.5–2.9 to 2.1–2.2 Å) correlated to a strong O=U=O bending angle (until 147° for one site). TRLFS and Raman experiments were carried out to check the validity of the computational results. Raman results supported the bidentate adsorption mode, and TRLFS measurements provided evidence that there was only one type of adsorption site upon the surface. Moreover, the formation of inner-sphere complexes was also confirmed, in agreement with DFT results. Indeed, these calculations show and confirm clearly that the displacement of water molecules seems always the mechanism involved, leading to an inner-sphere uranyl surface complex, whatever the mineral substrate reported in the literature.

Finally, interactions between uranyl ions and the hydrated Ni(111) surface model was studied. Two surface complexes were suggested: (i) the first one was an outer-sphere complex. Uranyl cation was adsorbed almost parallel to the surface, interacting by means of hydrogen bonds by sharing two water molecules of its first hydration shell with four water hexamers; (ii) uranyl rod adsorbed perpendicularly to the surface for the second complex, therefore leading to a surface complex possessing a strong covalent Ni-O^-yl^ bond (d(Ni-O^-yl^)=1.92 Å). Moreover, it interacted with water molecules of a hexamer which plays the role of first hydration shell, which was accordingly made of six water molecules, one more than for free uranyl. Even though the second surface complex was energetically the most stable one, the needed activation energy to reach it should be certainly too important to make this type of adsorption mode possible. An adsorption with an outer sphere mechanism should thus be more realistic on the hydrated Ni(111) face.

This study clearly showed that the solvent as well as the possible surface deprotonation have a great contribution to the uranyl adsorption process. Therefore, to complete this first study, it will be important to perform a higher level of calculation. As it was shown by a previous study [84] that DFT can provide accurate results for hydrogen bonds in liquid water, DFT molecular dynamics (CPMD) are being performed in our laboratory. Indeed, even though calculated structural parameters and interaction energies obtained from the static optimizations in vacuum condition were in good agreement with experimental results, the DFT molecular dynamics simulations will give a more complex and more detailed picture of the adsorption processes. This new approach will allow us: (i) to take into account the solvent effect which could favour or not the inner or outer sphere mechanism, (ii) to study the possible deprotonation of the surface sites during the dynamic process of the adsorption and (iii) to introduce temperature effect.

## Figures and Tables

**Table 1.** Calculated and experimental gibbsite bulk parameters.

**Parameter**	**This work^a^**	**Calculated^b^**	**Experimental^c^**
a (Å)	8.736	8.765	8.684
b (Å)	5.099	5.083	5.078
c (Å)	9.628	9.594	9.736
β (°)	92.83	92.63	94.54
V (Å^3^)	428.4	427.0	428.0

^(a)^ Veilly *et al.* [41]

^(b)^ Digne *et al.*[42] ;

^(c)^ Saalfeld *et al.* [39].

**Table 2.** Destabilization energies per dissociated water molecule as a function of the molecular (M) / dissociated (D) ratio (in eV). Percentage of dissociated water molecules was also reported.

**M / D** **Percentage**	**6 / 0** **0%**	**5 / 1** **17%**	**4 / 2** **33%**	**3 / 3** **50%**	**2 / 4** **66%**	**1 / 5** **83%**	**0 / 6** **100%**
$E_{\mathrm{destab}}^{\mathrm{average}}$	0.00	−0.03	−0.02	−0.06	−0.08	−0.11	−0.13
$E_{\mathrm{destab}}^{n}$	0.00	−0.03	−0.01	−0.14	−0.13	−0.23	−0.25

**Table 3.** Adsorption energies E_ads_ (in eV). Results from previous works are also summarized. See Figure 6 for notations.

	***E_ads_***	***ΔE_ads_***	***E_ads_* GGA [61]**	***E_ads_* exp.[62]**	***E_ads_* exp.[63]**	***E_ads_* exp.[64]**
**(1) on top**	**−0.34**	**0**	**−0.25**	**−0.42**	**−0.48**	**−0.53**
(2) fcc	−0.24	+0.10	–	–	–	–
(3) bridge	−0.18	+0.16	−0.10	–	–	–
(4) hcp	−0.14	+0.20	–	–	–	–

**Table 4.** Optimised geometries for the three bidentate adsorption sites (distances are in Å, bond angles in degree). Relative energies are meV. The most stable structure was taken as reference energy.

	***bb***	***bt***	***tt***	**EXAFS^e^**
U=O	1.91	1.90	1.86	1.78 ± 0.02
O=U=O	166.1	172.4	176.1	180
U–O_surface_	2.30	2.21^a^ / 2.28^b^	2.17	2.31 ± 0.02
U–O_water_	2.60^c^	2.62^c^	2.64^c^	2.46 ± 0.02
E_relative_^d^	0.0	85	175	

^a,b^ bond lengths with the bridging and the terminal oxygen atoms, respectively.

^c^ Average distance from the three bond lengths.

^d^ The *bb* structure is taken as reference because it was the most stable.

^e^ Experimental results from Den Auwer *et al.* [15].

**Table 5.** Main characteristics of the adsorbed complexes with respect to the surface protonation state. Distances are in Å, bond angles in degree and ΔE in eV. The most stable structure was taken as reference energy.

	**d(U-O_surf_)**	**d(U=O^-yl^)**	**d(O^-yl^••••H)**	**Θ(O=U=O)**	**ΔE**
***Protonated sites***					
Site I	2.61–2.70	1.88–1.96	1.81-1.82-2.32	169.6	0.28
Site II	2.53–2.87	1.92–1.94	1.71-1.75-1.79	176.0	0
Site III	2.60–2.70	1.92–1.95	1.61-1.72-2.32	168.4	0.24

***Deprotonated sites***					
Site I	2.11–2.21	1.87–1.92	1.95-2.04-2.31	146.9	0.58
Site II	2.10–2.24	1.87–1.89	1.74-1.77-1.83	162.9	0
Site III	2.12–2.17	1.90–1.92	1.69-1.79-2.08	150.8	0.40

**Table 6.** Adsorption energies of the uranyl ion on the dry Ni(111) face. Energies are in eV. To visualize the top, hcp, fcc and bridge adsorption sites, see Figure 6.

	**(1) top**	**(2) hcp**	**(3) bridge**	**(4) hcp**
*E* (eV)	−8.89	−8.66	−8.62	−8.03
*ΔE* (eV)	0.00	+0.23	+0.27	+0.86
